# *L3MBTL4* methylation is a sensitive marker of DNA-PK inhibitor in pancreatic cancer

**DOI:** 10.37349/etat.2026.1002382

**Published:** 2026-07-23

**Authors:** Yuanxin Yao, Yuan Li, Aiai Gao, Cheng Zhu, Ruijie Wang, Yazhuo Li, Xiaomo Su, Meiying Zhang, Mingzhou Guo

**Affiliations:** National University of Singapore, Singapore; ^1^Department of Gastroenterology and Hepatology, Chinese PLA General Hospital, Beijing 100853, China; ^2^School of Medicine, Nankai University, Tianjin 300071, China; ^3^National Key Laboratory of Kidney Diseases, Chinese PLA General Hospital, Beijing 100853, China

**Keywords:** *L3MBTL4*, DNA methylation, synthetic lethality, DNA damage repair, NU7441, pancreatic cancer

## Abstract

**Aim:**

The purpose is to explore the mechanism and new therapeutic strategy of lethal 3 malignant brain tumor like 4 (*L3MBTL4*) gene in pancreatic ductal adenocarcinoma (PDAC).

**Methods:**

Immunoprecipitation, siRNA knockdown, immunohistochemistry, homologous recombination (HR) and non-homologous end joining (NHEJ) reporter assays, comet assays, and a xenograft mouse model were employed.

**Results:**

*L3MBTL4* was methylated in 16.3% (7/43) of intraductal papillary mucinous neoplasms, 19.0% (4/21) of mucinous cystic neoplasm, and 28.2% (84/298) of PDAC, and its expression was regulated by promoter region methylation. *L3MBTL4* methylation was significantly associated with tumor differentiation and tumor size. The expression of *L3MBTL4* inhibited cell proliferation, colony formation, and induced apoptosis and G1/S phase arrest. L3MBTL4 activated ATM/CHK2 and inhibited NHEJ signaling by interacting with Ku70. Loss of *L3MBTL4* increased the sensitivity of PDAC cells to NU7441 both in vitro and in vivo.

**Conclusions:**

*L3MBTL4* is a new component of NHEJ signaling and epigenetic silencing of *L3MBTL4* sensitizes PDAC cells to DNA-PK inhibitors, providing a potential new therapeutic strategy.

## Introduction

Pancreatic ductal adenocarcinoma (PDAC) is becoming the third leading cause of cancer-related mortality globally [1]. In addition to age, pancreatitis and inheritable genetic factors, cigarette smoking, obesity, diabetes, and alcohol intake are regarded as important modifiable risk factors [2]. Even though, new approaches and models have been extensively explored, the early detection and poor prognosis remain unimproved [3–7]. Most patients are unresectable at the time of diagnosis, and only 15–20% are eligible for surgery [8]. However, even among surgically resected patients, 75% will experience recurrence within 2 years [9]. Even though chemotherapeutic regimen of the standard-of-care has been improved, it is still a challenge for curing PDAC. The application of “synthetic lethality” concept has propelled the identification of more DNA damage repair (DDR) gene mutations for cancer-targeting therapy [10–13]. PDAC patients with homologous recombination (HR) gene mutations (*BRCA1/2* and *PALB2*) have improved progression-free survival and overall survival (OS) after platinum-based chemotherapy and poly (ADP-ribose) polymerase inhibitor treatment [14]. Since the discovery of “synthetic lethality” principle, it has profoundly influenced our understanding of DDR, cancer development, and treatment [15, 16]. Most studies have focused on the genetic contribution to synthetic lethal effects. “BRCAness” was introduced to describe the defects of other HR genes with the phenotype mimicking BRCA1/2 loss [17–19]. Increasing evidence demonstrates that epigenetic programming drives tumor progression either independently or in cooperation with genetic deficiency [20, 21]. In contrast to genetic mutations, epigenetic alterations may be reversible by targeting epigenetic modifiers (reader, writer, and eraser) with small molecule inhibitors [22–24]. However, these key enzymes play important roles in both normal and cancer cells, making them nonspecific to cancer cells. Thus, this kind of epigenetic targeting therapy may activate unnecessary signaling pathways and further cause other diseases. A recent report provided a vivid clinical clue that a second tumor emerged during the treatment of ovarian cancer with tazemetostat, an enhancer of zeste homolog 2 (EZH2) inhibitor [25]. The results demonstrate that classical epigenetic therapy is a double-edged sword. Therefore, harnessing the principle of “synthetic lethality” and the aberrant alterations of epigenetics may develop more precise targeting therapeutic strategies, without hurting normal cells [20, 21, 26–29]. To this end and to widen its application, more epigenetic abnormality markers for cell fate-determination or DDR-related regulators need to be identified in cancers to look for novel therapeutic targets and overcome drug-resistance.

The malignant brain tumor (MBT) repeat is a structural motif, comprising approximately 100 amino acid residues. The MBT domain is conserved from *Caenorhabditis elegans* to humans [30, 31]. There are 9 MBT domain-containing proteins, which play crucial roles in chromatin remodeling, gene expression regulation, and DDR. Lethal 3 malignant brain tumor like 1 (*L3MBTL1*) and *L3MBTL2* were discovered to promote DNA double-strand breaks (DSBs) repair [32, 33]. *L3MBTL4* is a new member of this family, and the levels of its mRNA were reduced in breast cancer [34]. While the biological function and mechanism of *L3MBTL4* are unclear.

## Materials and methods

### PDAC cells and primary cancer samples

Cells were established previously from primary PDAC, including PANC10.05, BxPC3, MIA PaCa-2, PANC3.11 and SW1990. The STR reports of PANC10.05, BxPC3, MIA PaCa-2 and SW1990 cell lines are available (identified by the BIOWING company). PANC3.11 cells were established by Elizabeth M. Jaffee from Johns Hopkins University and have been published [35, 36]. Mycoplasma contamination testing was performed for these cell lines.

Primary cancer samples were collected at the Chinese PLA General Hospital, which were comprised of intraductal papillary mucinous neoplasm (IPMN, 43 cases), mucinous cystic neoplasm (MCN, 21 cases), and PDAC (298 cases). All patients were not subjected to chemo-radiotherapy before surgery. Tumors were classified according to tumor-node-metastasis (TNM) staging (AJCC 2019). The procedures were performed in accordance with the Declaration of Helsinki. The study was approved by the Institutional Review Board of the Chinese PLA General Hospital, and informed consent was obtained from all patients (Approval No. 20090701-015, 20160204-26 and S2025-791-01).

### RNA and DNA preparation, PCR amplification and 5-aza-2’-deoxycytidine (5-aza) treatment

DNA was prepared with phenol-chloroform and modified following previous approaches [37]. Total RNA preparation and cDNA synthesis followed the manufacturer’s instructions (#K1691, Thermo Scientific, USA). Demethylating reagent, 5-aza (2 μmol/L), was utilized to induce re-expression of epigenetically silenced genes (#A3656, Sigma-Aldrich, USA). The primers are listed in Table S1.

### Immunohistochemistry (IHC)

Antibodies used for IHC are shown in Table S2. Paraffin samples were stained according to a previous description. The staining score was evaluated following the German semiquantitative scoring criteria [38].

### Establishing L3MBTL4 stably expressed cells

The coding region of *L3MBTL4* (referenced as NM_173464) was inserted into pCDH-CMV-MCS-puro plasmid. *L3MBTL4* expressing or empty vectors were transfected into HEK293 cells using Lipofectamine 3000 Reagent (Invitrogen, USA). Lentiviral supernatant was then added to the RPMI-1640 medium (31800089, Gibco, USA) containing 10% fetal bovine serum (900-108, GEMINI, USA) and 1% penicillin/streptomycin solution (BL505A, Biosharp, China). *L3MBTL4* expressing cells were selected by puromycin treatment at a concentration of 2.5μg/mL (BxPC3) and 1.5 μg/mL (MIA PaCa-2) for 3 days. Single-cell clones were screened by limited dilution in 96 well plates and validated using western blotting.

### 3-(4,5-dimethylthiazol-2-yl)-2,5-diphenyltetrazolium bromide (MTT), colony formation and flow cytometry assays

Cells were seeded in 96- well plates for 2 × 10^3^ BxPC3 and 2 × 10^3^ MIA PaCa-2 cells per well for MTT assay (#KGT5251, KeyGEN Biotech, China). Six-well plates were used to perform colony formation, with 1 × 10^3^ cells/well for 12 days. Cell cycle and apoptosis were analyzed by propidium iodide (PI) staining (#KGA512, KeyGEN Biotech, China) and Annexin V-FITC/PI Apoptosis Detection Kit (#KGA108, KeyGEN Biotech, China) with FACScan flow cytometer.

### Testing the efficiency of NU7441 in cisplatin treated PDAC cell models

The efficiency of NU7441 was tested by MTT assay. For 50% inhibitory concentration (IC_50_) analysis, PDAC cells were seeded in 96-well plates at 2 × 10^3^ per well, and the cell viability was evaluated by the OD value after treatment for 48 h. The sensitivity of PDAC cells to NU7441 (#HY-11006, MCE, USA) was assessed by colony formation assay. Cells were seeded with 2000 each well and treated with 0.01 μM cisplatin (#S1166, Selleck, USA) and 0.5 μM NU7441 for 48 h. Thereafter, the medium was changed to RPMI-1640 containing 10% fetal bovine serum and 1% penicillin/streptomycin solution, and the cells were grown for 10 days.

### siRNA knockdown, western blot and immunoprecipitation (IP) assays

RNAiMax reagent was utilized for *L3MBTL4* knockdown. The procedure was performed according to instructions (#13778150, Invitrogen, USA). The sequences of the siRNAs are shown in Table S1 (JTS Scientific, China). Antibodies used for western blotting and IP are listed in Table S2. Mass spectrometry technique was used to analyze the components of the interacting complex.

### HR and non-homologous end joining (NHEJ) reporter and comet assays

HR and NHEJ reporter assays were followed as in a previous study [29]. The U2OS cells were identified by the Procell Life Science company, and the STR report of U2OS cells is available. *L3MBTL4* silenced or re-expressed BxPC3 and MIA PaCa-2 cells were grown in 6-well plates and transfected with 1 μg of pCVL Traffic Light Reporter 1.1 (Sce target) Ef1a Puro plasmids and 1 μg of I-SceI plasmids. The cells were harvested for FACS analysis after growing for 48 h, and the data were analyzed using FlowJo (BD Biosciences, USA). The experiments were performed in triplicate.

To evaluate the impact of *L3MBTL4* on DDR, the alkaline comet electrophoresis method was employed [29, 39]. DNA damaged PDAC cell models was induced by cisplatin at 1 μM and 0.5 μM for BxPC3 and MIA PaCa-2 cells, respectively. The tail moments were quantified by the Comet Score (version2.0, Tritek Corp.). Each group included 100 cells.

### Silencing of *L3MBTL4* sensitizes PDAC cell xenograft to DNA-PK inhibitor

A PDAC cell xenograft mouse model was employed for evaluating the influence of *L3MBTL4* methylation in DNA-PK inhibitor sensitivity. *L3MBTL4* silenced or re-expressed MIA PaCa-2 cells (4 × 10^6^ cells) were injected subcutaneously into four-week-old female BALB/c nude mice (purchased from SPF company, Beijing, China). The tumor volume was calculated following a previous study [28]. When the tumor volume reached about 100 mm^3^, the mice were randomly divided into four groups (each group with 6 mice), including the control group, cisplatin group (2 mg/kg), NU7441 group (10 mg/kg), and cisplatin combined with NU7441 group (2 mg/kg of cisplatin and 10 mg/kg of NU7441). Cisplatin and NU7441 were administered twice a week intraperitoneally for two weeks. Tumors were measured for 21 days, 3 days each time. Thereafter, the mice were euthanized using cervical dislocation by experienced personnel in strict compliance with the Guide for the Care and Use of Laboratory Animals. The animal experiments were performed according to the guidelines approved by the Animal Ethics Committee of the Chinese PLA General Hospital (Approval NO. 2021-X17-44).

### Statistical methods

GraphPad Prism 8.0 software (GraphPad Software Inc., CA, USA) was utilized for statistical analysis. The associations between *L3MBTL4* methylation and clinicopathological factors were analyzed by chi-square test. Kaplan–Meier plots and the log-rank test were used to estimate the OS. Univariate and multivariate Cox regression analysis were applied to evaluate the influencing factors of survival time. The difference of the OD values was analyzed by repeated measures ANOVA and Bonferroni’s multiple comparisons test in *L3MBTL4* unexpressed and re-expressed PDAC cells. The difference between two groups was assessed by the Student’s t-test. *P* < 0.05 was regarded as statistically significant.

## Results

### *L3MBTL4* is silenced in human PDAC

To explore the possibility of epigenetic regulation of *L3MBTL4*, the RNA expression and DNA methylation data of PDAC were obtained from GTEx and The Cancer Genome Atlas (TCGA) databases (http://xena.ucsc.edu/). The levels of L3MBTL4 mRNA were significantly lower in PDAC samples than in normal pancreatic tissue (*P* < 0.001, Figure S1A and Supplementary material). The expression of *L3MBTL4* was reversely associated with the methylation of CpG sites in the promoter region (cg14693194, cg26120251, cg08007465, cg02731042 and cg12924825, all *P* < 0.05, Figure S1B–1C and Supplementary material). These findings hint that *L3MBTL4* is possibly regulated by DNA methylation.

**Figure 1:**
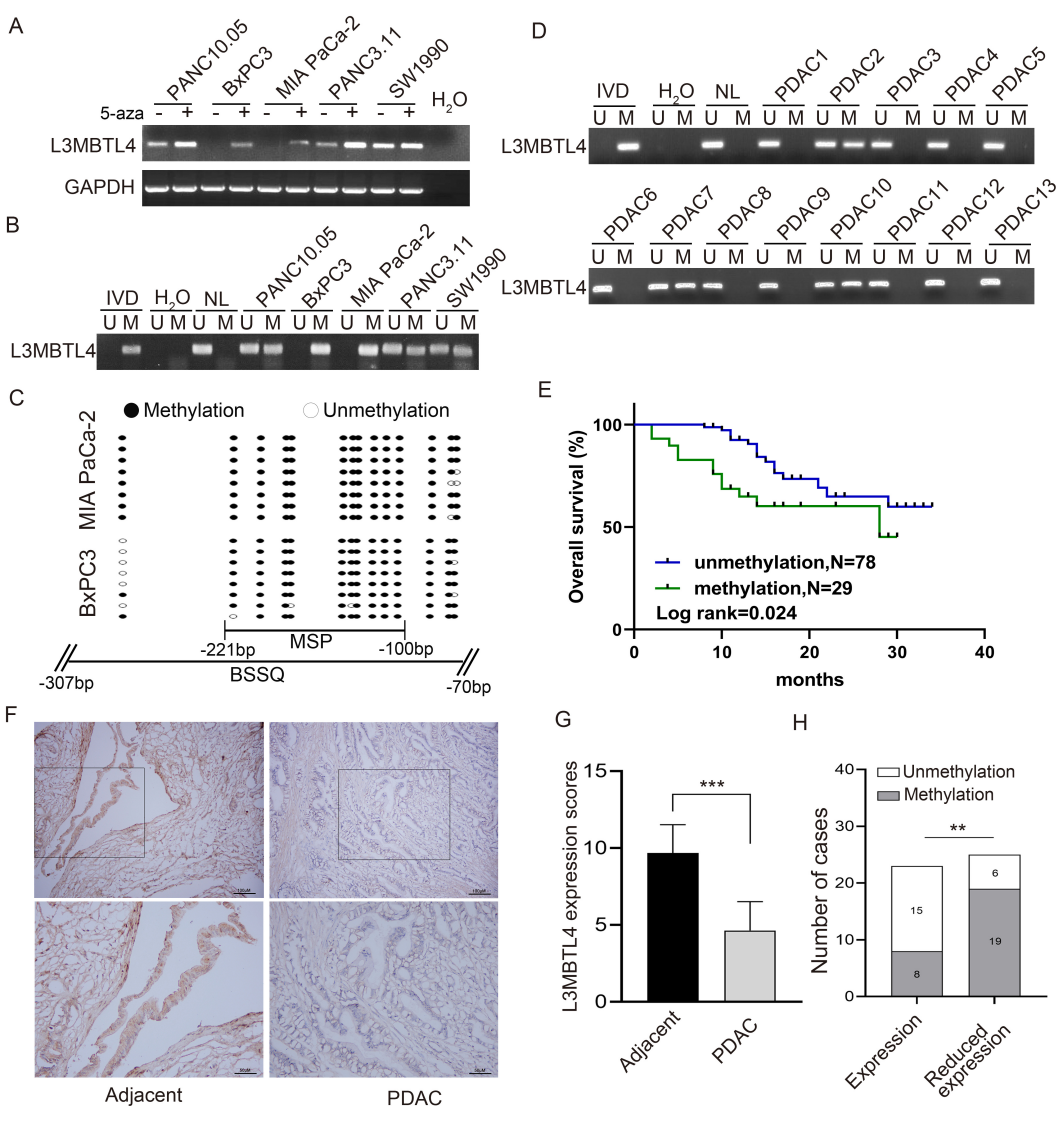
**The expression and methylation status of lethal 3 malignant brain tumor like 4 (*L3MBTL4*) in pancreatic ductal adenocarcinoma (PDAC) cells and tissue samples.** (**A**) RT-PCR results of *L3MBTL4* in PDAC cells. 5-aza: 5-aza-2’-deoxycytidine; GAPDH: internal control; (-): absence of 5-aza; (+): presence of 5-aza. (**B**) Methylation specific PCR (MSP) results of *L3MBTL4* in PDAC cells. U: unmethylation alleles; M: methylation alleles; IVD: in vitro methylated DNA, serves as a methylation control; NL: normal peripheral lymphocytes DNA, serves as an unmethylation control. (**C**) Bisulfite sequencing (BSSQ) results of *L3MBTL4*. Filled circles: methylated CpG sites; open circles: unmethylated CpG sites. (**D**) Representative MSP results of *L3MBTL4* in PDAC samples. (**E**) Overall survival (OS) for *L3MBTL4* methylated and unmethylated PDAC patients (Kaplan–Meier plots). (**F**) Representative immunohistochemistry (IHC) staining of L3MBTL4 in PDAC and adjacent noncancerous tissue samples. Scale bar: 100 μM (top); 50 μM (bottom). (**G**) L3MBTL4 IHC score (Wilcoxon-test analysis). (**H**) Bar diagram indicating an inverse relationship between L3MBTL4 expression levels and DNA methylation status (chi-square test analysis). **P < 0.01, ***P < 0.001.

To validate the promoter region methylation regulating *L3MBTL4* expression, PDAC cells were detected with RT-PCR and methylation specific PCR (MSP). *L3MBTL4* was not expressed in BxPC3 and MIA PaCa-2 cells, while reduced expression was exhibited in PANC10.05, PANC3.11 and SW1990 cells (Figure 1A). Complete methylation was observed in BxPC3 and MIA PaCa-2 cells, and partial methylation emerged in PANC10.05, PANC3.11 and SW1990 cells (Figure 1B). The reverse association of *L3MBTL4* expression and methylation was shown in PDAC cells. The result was verified by inducing its expression with 5-aza (Figure 1A). The methylation density and the efficiency of MSP primers were verified by bisulfite sequencing (BSSQ) in MIA PaCa-2 and BxPC3 cells (Figure 1C).

Thereafter, the methylation status of *L3MBTL4* was detected in IPMN, MCN and PDAC and it was methylated in 16.3% (7/43) of IPMN, 19.0% (4/21) of MCN, and 28.2% (84/298) of PDAC (Figure 1D). *L3MBTL4* methylation was significantly associated with tumor differentiation (*P* < 0.001) and tumor size (*P* < 0.01, Table 1), indicating that *L3MBTL4* methylation is increased with tumor progression. *L3MBTL4* methylation was associated with poor OS (*P* < 0.05, Figure 1E) and was an independent prognostic factor for poor OS in 107 cases of follow up data available patients (*P* < 0.05, Table 2).

**Table 1:** The association of lethal 3 malignant brain tumor like 4 (*L3MBTL4*) methylation and clinical factors in pancreatic ductal adenocarcinoma (PDAC).

**Variables**	**Number**	**Methylation status**		***P* value**
		**Unmethylation** ***N* = 214**	**Methylation** ***N* = 84**	
Sex				0.2841
Female	108	82	26	-
Male	190	132	58	-
Age (years)				0.7346
≤ 50	52	36	16	-
> 50	246	178	68	-
Smoking				0.5107
No	183	134	49	-
Yes	115	80	35	-
Alcohol				0.7963
No	164	119	45	-
Yes	134	95	39	-
Differentiation				< 0.001^***^
Well or moderate	140	119	21	-
Poor	158	95	63	-
Nerve invasion				0.1183
No	86	56	30	-
Yes	212	158	54	-
Tumor size (cm)				0.0066^**^
≤ 4	237	179	58	-
> 4	61	35	26	-
Lymph node metastasis				0.4207
Negative	195	143	52	-
Positive	103	71	32	-
TNM stage				> 0.9999
Stage I–II	269	193	76	-
Stage III–VI	29	21	8	-

*P* values are obtained from the chi-square test. **P < 0.01, ***P < 0.001. TNM: tumor-node-metastasis.

**Table 2:** Univariate and multivariate analysis of lethal 3 malignant brain tumor like 4 *(L3MBTL4*) methylation status with overall survival in pancreatic ductal adenocarcinoma (PDAC).

**Clinical parameter**	**Univariate analysis**		**Multivariate analysis**	
	**HR (95%CI)**	***P* value**	**HR (95%CI)**	***P* value**
Gender (male vs. female)	1.189 (0.548, 2.578)	0.662	-	-
Age (> 50 vs. ≤ 50 years)	1.095 (0.416, 2.885)	0.854	-	-
Tumor size (> 4 vs. ≤ 4 cm)	1.867 (0.842, 4.141)	0.124	-	-
Differentiation (poor vs. well or moderate)	0.579 (0.270, 1.241)	0.160	-	-
Lymph node metastasis (positive vs. negative)	2.557 (1.206, 5.422)	0.014^*^	2.435 (1.146, 5.173)	0.021^*^
Nerve invasion (yes vs. no)	0.881 (0.387, 2.003)	0.762	-	-
TNM stage (III–VI vs. I–II)	1.504 (0.521, 4.340)	0.450	-	-
*L3MBTL4* (methylation vs. unmethylation)	2.296 (1.084, 4.862)	0.030^*^	2.176 (1.024, 4.624)	0.043^*^
Smoking (yes vs. no)	1.377 (0.649, 2.921)	0.404	-	-
Alcohol (yes vs. no)	0.582 (0.268, 1.264)	0.171	-	-

HR: hazard ratio; TNM: tumor-node-metastasis. *P < 0.05.

Then, the expression and methylation status of *L3MBTL4* were analyzed. L3MBTL4 was stained in both nucleus and cytoplasm. The staining score of L3MBTL4 was lower in cancer tissue samples compared to adjacent normal tissues (Figure 1F and 1G, *P* < 0.001). The high level of L3MBTL4 was revealed in 23 cases of PDAC tissue samples, among which 8 cases were methylated (34.8%). Reduced level of L3MBTL4 was observed in 25 cases of PDAC, among which 19 cases were methylated (76.0%). Methylation of *L3MBTL4* was associated with its low-level expression, implying *L3MBTL4* methylation regulates its expression (Figure 1H, *P* < 0.01).

### *L3MBTL4* inhibits PDAC cell proliferation, induces apoptosis and G1/S arrest

**Figure 2:**
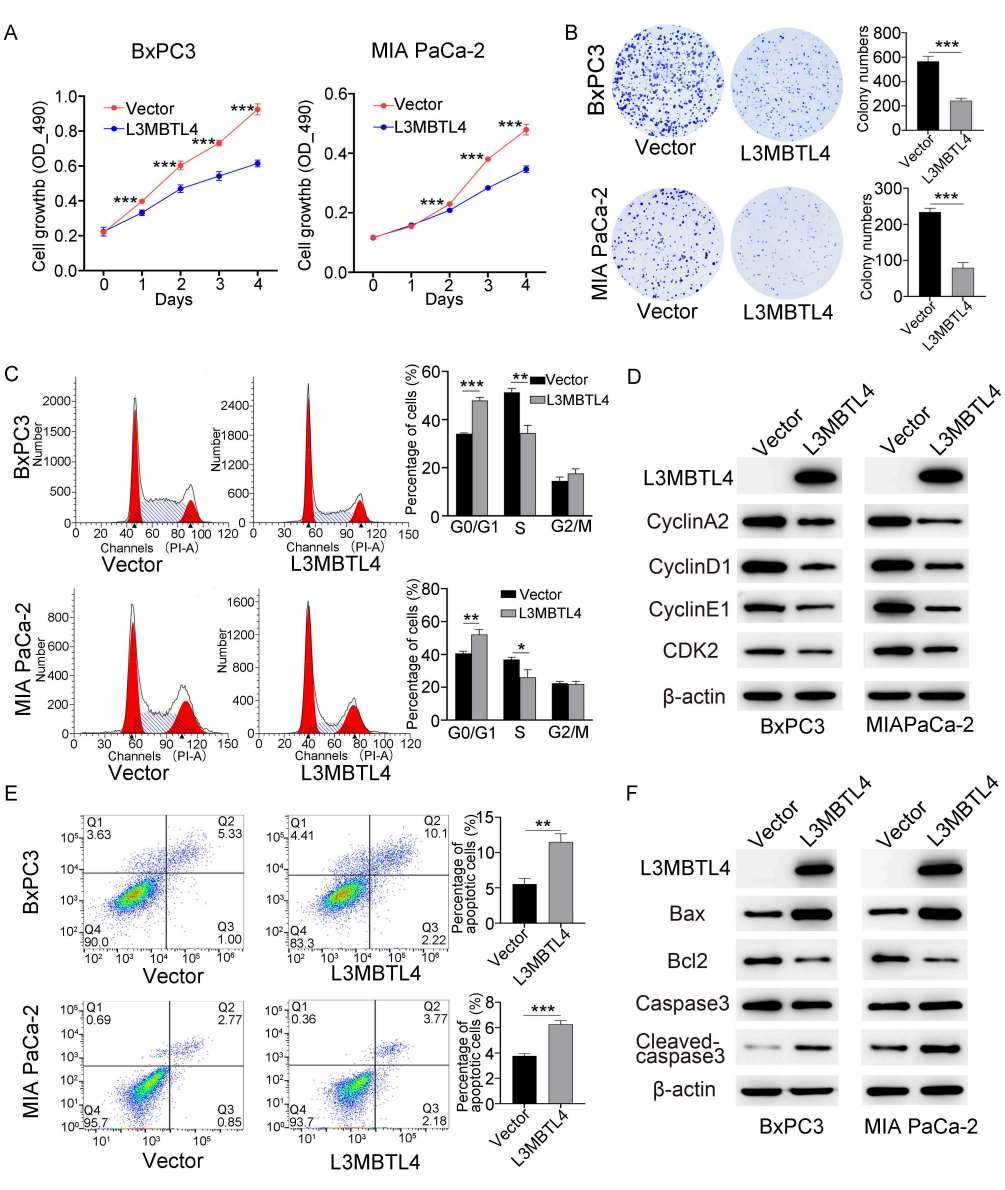
**Effect of lethal 3 malignant brain tumor like 4 (*L3MBTL4*) on pancreatic ductal adenocarcinoma (PDAC) cell proliferation, colony formation, cell cycle, and apoptosis.** (**A**) The OD values in *L3MBTL4* silenced and re-expressed PDAC cells in different time, analyzed by repeated measures ANOVA and Bonferroni’s multiple comparisons test. (**B**) Representative colony formation results. (**C**) Representative results of cell phase distribution. The bar diagram represents the percentage. (**D**) Western blot showing the effects of L3MBTL4 on the expression levels of G1/S regulators. (**E**) Apoptosis results. (**F**) Western blot results of apoptosis related proteins. Vector: empty vector control; *L3MBTL4*: *L3MBTL4* expressing vector. *P < 0.05, **P < 0.01, ***P < 0.001

The viability of PDAC cells with or without *L3MBTL4* expression was evaluated using MTT assay. The OD values were 0.92 ± 0.03 vs. 0.61 ± 0.02 and 0.48 ± 0.02 vs. 0.35 ± 0.01 in BxPC3 and MIA PaCa-2 cells before and after restoration of *L3MBTL4* expression for 96 hours, respectively (both *P* < 0.001, Figure 2A). The OD value was reduced by *L3MBTL4*, indicating an inhibitory effect on cell proliferation.

Colony formation assay was performed to evaluate the role of *L3MBTL4* in PDAC cell growth. The clone numbers were 566.0 ± 41.4 vs. 243.7 ± 19.4 and 234.3 ± 10.3 vs. 79.7 ± 14.4 in *L3MBTL4* unexpressed and re-expressed BxPC3 and MIA PaCa-2 cells, respectively (both *P* < 0.001, Figure 2B). *L3MBTL4* reduced PDAC cell colon number, demonstrating the inhibitory role of *L3MBTL4* in PDAC cells.

The effect of *L3MBTL4* on the cell cycle was assessed with flow cytometry. The percentages of G0/G1 phase cells were 34.2 ± 0.3% vs. 48.0 ± 1.2% (*P* < 0.001) and 40.6 ± 1.3% vs. 52.1 ± 3.1% (*P* < 0.01) in *L3MBTL4* unexpressed and re-expressed BxPC3 cells and MIA PaCa-2 cells, respectively. The percentages were 51.4 ± 1.6% vs. 34.4 ± 3.2% (*P* < 0.01) and 36.9 ± 1.4% vs. 26.0 ± 4.7% (*P* < 0.05) in the S phase, respectively. The ratios of G2/M phase cells were 14.5 ± 1.7% vs. 17.6 ± 1.9% and 22.4 ± 1.2% vs. 21.9 ± 1.7% before and after re-expression of *L3MBTL4* in BxPC3 cells and MIA PaCa-2 cells, respectively (Figure 2C). These results indicate that *L3MBTL4* re-expression induces G1/S arrest in PDAC cells. The levels of G1/S checkpoint related proteins were analyzed. The levels of Cyclin A2, Cyclin D1, Cyclin E1, and CDK2 proteins were reduced by *L3MBTL4* expression, validating the effect of *L3MBTL4* on cell cycle (Figure 2D).

The role of *L3MBTL4* in apoptosis was measured by flow cytometry. The percentages of apoptotic cells were as follows: 5.5 ± 0.8% vs. 11.5 ± 1.2% (*P* < 0.01) and 3.8 ± 0.2% vs. 6.3 ± 0.3% (*P* < 0.001) in *L3MBTL4* unexpressed and re-expressed BxPC3 and MIA PaCa-2 cells, respectively (Figure 2E). These results suggest that *L3MBTL4* induces PDAC cell apoptosis. Representative apoptotic markers were detected by western blot. *L3MBTL4* reduces the level of Bcl2 and increases the levels of Cleaved-caspase3 and Bax (Figure 2F), further verifying its role in inducing apoptosis.

### L3MBTL4 is involved in DDR by interacting with Ku70

**Figure 3:**
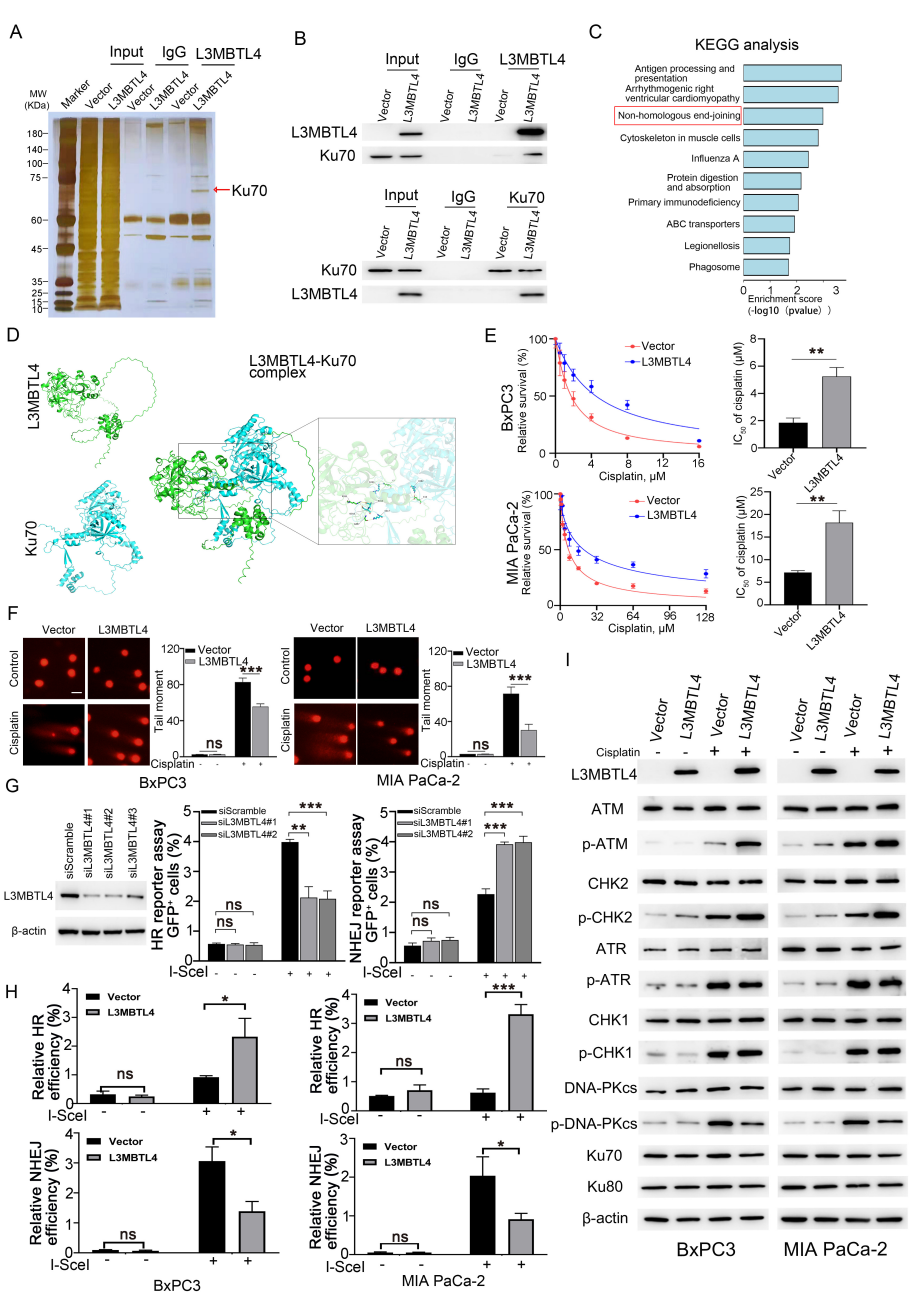
**Lethal 3 malignant brain tumor like 4 (*L3MBTL4*) activates ATM/CHK2 signaling and inhibits non-homologous end joining (NHEJ) signaling.** (**A**) IP assay and silver staining. Red arrow: differential band. IgG: negative control. (**B**) Validation of the interaction between L3MBTL4 and Ku70. (**C**) KEGG pathway analysis of L3MBTL4 binding proteins from mass spectrometry assay. (**D**) The interaction of L3MBTL4 and Ku70 predicted by AlphaFold3 software. (**E**) Evaluation of the IC_50_ of cisplatin in pancreatic ductal adenocarcinoma (PDAC) cells. (**F**) Comet assay of PDAC cells before and after cisplatin treatment. (**G**) The knockdown efficiency of siRNA targeting *L3MBTL4* in U2OS cells (left). Homologous recombination (HR) and NHEJ efficiency were evaluated in U2OS-DR-GFP and U2OS-EJ5 cells before and after *L3MBTL4* knockdown, respectively (right). (**H**) HR and NHEJ efficiencies were evaluated in BxPC3 and MIA PaCa-2 cells with or without L3MBTL4 expression. (**I**) Effects of *L3MBTL4* on ATM/CHK2, ATR/CHK1, and NHEJ signaling pathways under the treatment of cisplatin for 48 h. cisplatin (-): without cisplatin treatment; cisplatin (+): with cisplatin treatment. *P < 0.05, **P < 0.01, ***P < 0.001.

To understand the mechanism of *L3MBTL4* in PDAC, IP and mass spectrometry techniques were utilized. Ku70 was discovered with the highest score in the complex (Figure 3A). This finding was validated by reciprocal IP and western blot (Figure 3B). KEGG pathway analysis was performed to analyze the binding proteins, and NHEJ components were uncovered to be an important part (Figure 3C). Then, the interaction of L3MBTL4 and Ku70 was tested by AlphaFold3 (Figure 3D).

Ku70 is a key component of NHEJ signaling. *L3MBTL1* and *L3MBTL2*, the family members of *L3MBTL4*, have been reported to participate in DDR [32, 33, 40]. DDR is a double-edged sword for carcinogenesis and cancer chemoradiotherapy. Next, we evaluated the impact of *L3MBTL4* on DDR in PDAC cells. In *L3MBTL4* silenced and re-expressed BxPC3 and MIA PaCa-2 cells, the IC_50_ values of cisplatin were 1.8 ± 0.4 µM vs. 5.3 ± 0.6 µM (*P* < 0.01) and 7.2 ± 0.4 µM vs. 18.2 ± 2.6 µM (Figure 3E, *P* < 0.01), respectively. These results demonstrated that *L3MBTL4* reduces the sensitivity of PDAC cells to cisplatin, reflecting the potential role of L3MBTL4 in DDR.

Thereafter, the effect of *L3MBTL4* on DSB repair was evaluated via a comet assay. In *L3MBTL4* silenced and re-expressed BxPC3 and MIA PaCa-2 cells, the tail moment was 83.0 ± 4.4 vs. 55.7 ± 3.1 (Figure 3F, *P* < 0.001) and 71.7 ± 7.6 vs. 30.3 ± 6.5 (*P* < 0.001), respectively. These results indicate that *L3MBTL4* is involved in DNA DSB repair.

### *L3MBTL4* activates ATM/CHK2 and inhibits NHEJ signaling

In mammalian cells, DSBs are the most severe DNA damage, as they may cause large chromosomal region loss, and DSBs are repaired predominantly by HR and NHEJ pathways. To better understand the mechanisms of *L3MBTL4* in DNA DSB repair, HR and NHEJ efficiency were measured by HR and NHEJ reporter assays. HR efficiency was significantly reduced by *L3MBTL4* knockdown in U2OS cells expressing DR-GFP, indicating that *L3MBTL4* promotes HR signaling (Figure 3G). The efficiency of NHEJ was significantly increased after knockdown of *L3MBTL4* in U2OS cells expressing EJ5-GFP, implying the inhibitory role of *L3MBTL4* in NHEJ signaling (Figure 3G). Thereafter, the role of *L3MBTL4* in DDR was validated in BxPC3 and MIA PaCa-2 cells (Figure 3H).

To further validate the roles of *L3MBTL4* in HR and NHEJ signaling, the important molecules of these pathways were detected. Increased p-ATM and p-CHK2 levels were observed after restoration of *L3MBTL4* expression in BxPC3 and MIA PaCa-2 cells, revealing the activating role of *L3MBTL4* in ATM pathway (Figure 3I). The impact of *L3MBTL4* on ATR/CHK1 signaling was also evaluated. However, no obvious changes were observed for the levels of p-ATR and p-CHK1 (Figure 3I). The inhibitory role of *L3MBTL4* in NHEJ pathway was verified by detecting the level of p-DNA-PKcs and an increased level of p-DNA-PKcs was observed in *L3MBTL4* unexpressed PDAC cells (Figure 3I).

### *L3MBTL4* defects increased the sensitivity of PDAC cells to DNA-PK inhibitor

**Figure 4:**
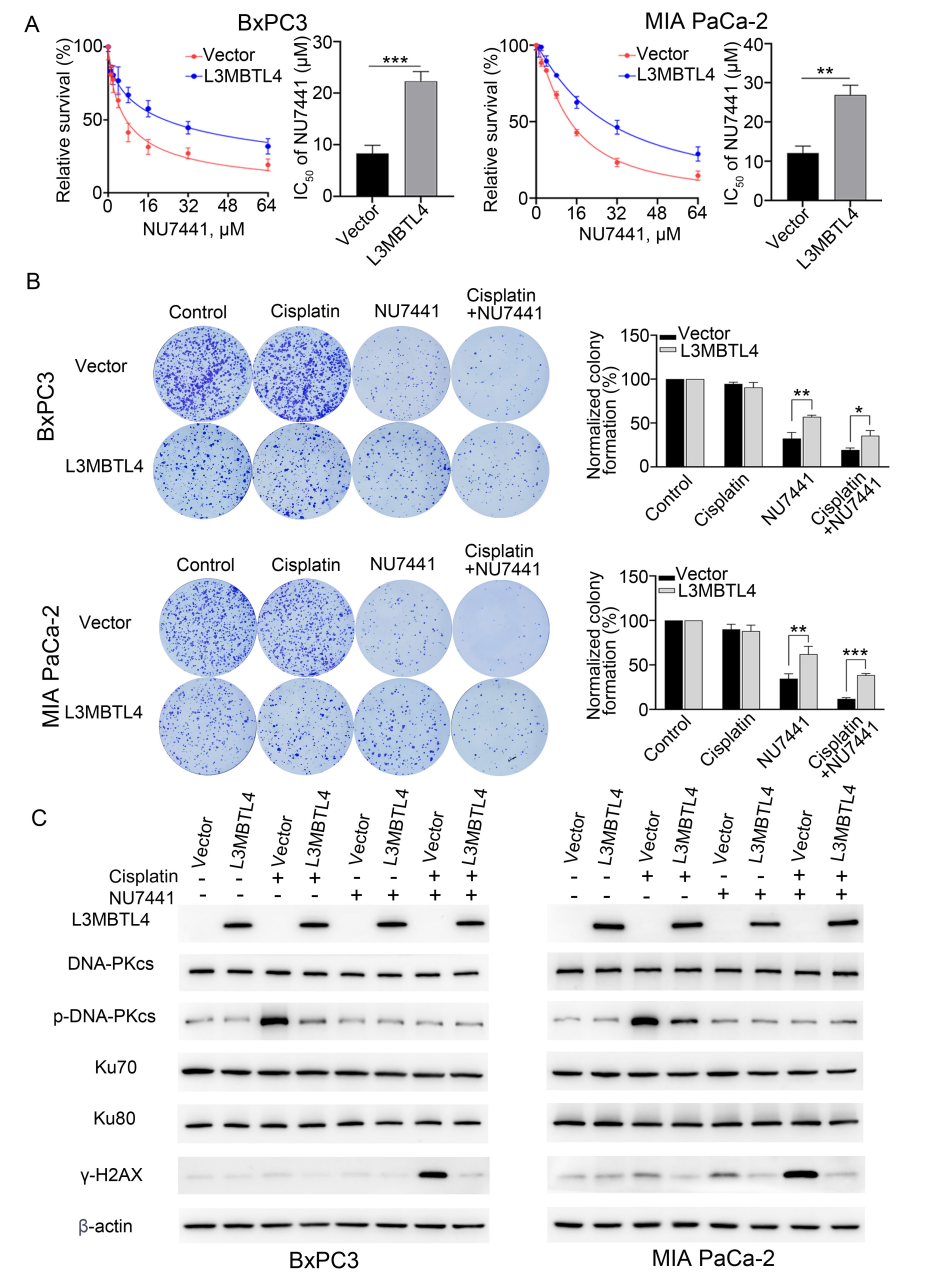
**Loss of lethal 3 malignant brain tumor like 4 (*L3MBTL4*) expression sensitized pancreatic ductal adenocarcinoma (PDAC) cells to NU7441.** (**A**) 50% inhibitory concentration (IC_50_) assay showing the sensitivity of NU7441 in PDAC cells under the treatment of low dose cisplatin. (**B**) Representative colony formation results showing the defect of *L3MBTL4* increases sensitivity to NU7441. (**C**) The levels of non-homologous end joining (NHEJ) signaling and γ-H2AX in cells under treatment with cisplatin and NU7441. *P < 0.05, **P < 0.01, ***P < 0.001.

To acquire more evidence that *L3MBTL4* is involved in NHEJ, an MTT assay was utilized to evaluate the IC_50_ of NU7441, a DNA-PK inhibitor, in PDAC cells. In BxPC3 and MIA PaCa-2 cells, the IC_50_ values were 8.3 ± 1.6 vs. 22.3 ± 1.9 µM (*P* < 0.001) and 12.1 ± 1.8 vs. 26.9 ± 2.5 µM (*P* < 0.01), without the expression or restoration of *L3MBTL4*, respectively (Figure 4A). These results demonstrate that loss of *L3MBTL4* expression sensitizes PDAC cells to DNA-PK inhibitors.

The low dose cisplatin induced DNA damage cell model was subsequently used to test the sensitivity of *L3MBTL4* silenced PDAC cells to DNA-PK inhibitors. Under cisplatin and 0.5 μM NU7441 treatment, the normalized colony efficiency was 19.1 ± 2.5% vs. 35.6 ± 5.8% (*P* < 0.05) and 11.7 ± 1.4% vs. 38.4 ± 1.9% (*P* < 0.001) without expression or re-expression of *L3MBTL4* in BxPC3 and MIA PaCa-2 cells (Figure 4B). These results suggested that loss of *L3MBTL4* expression sensitized PDAC cells to NU7441. This effect was further validated by detecting the levels of γ-H2AX (Figure 4C).

### Epigenetic silencing of *L3MBTL4* sensitizes MIA PaCa-2 cell xenografts to DNA-PK inhibitor

**Figure 5:**
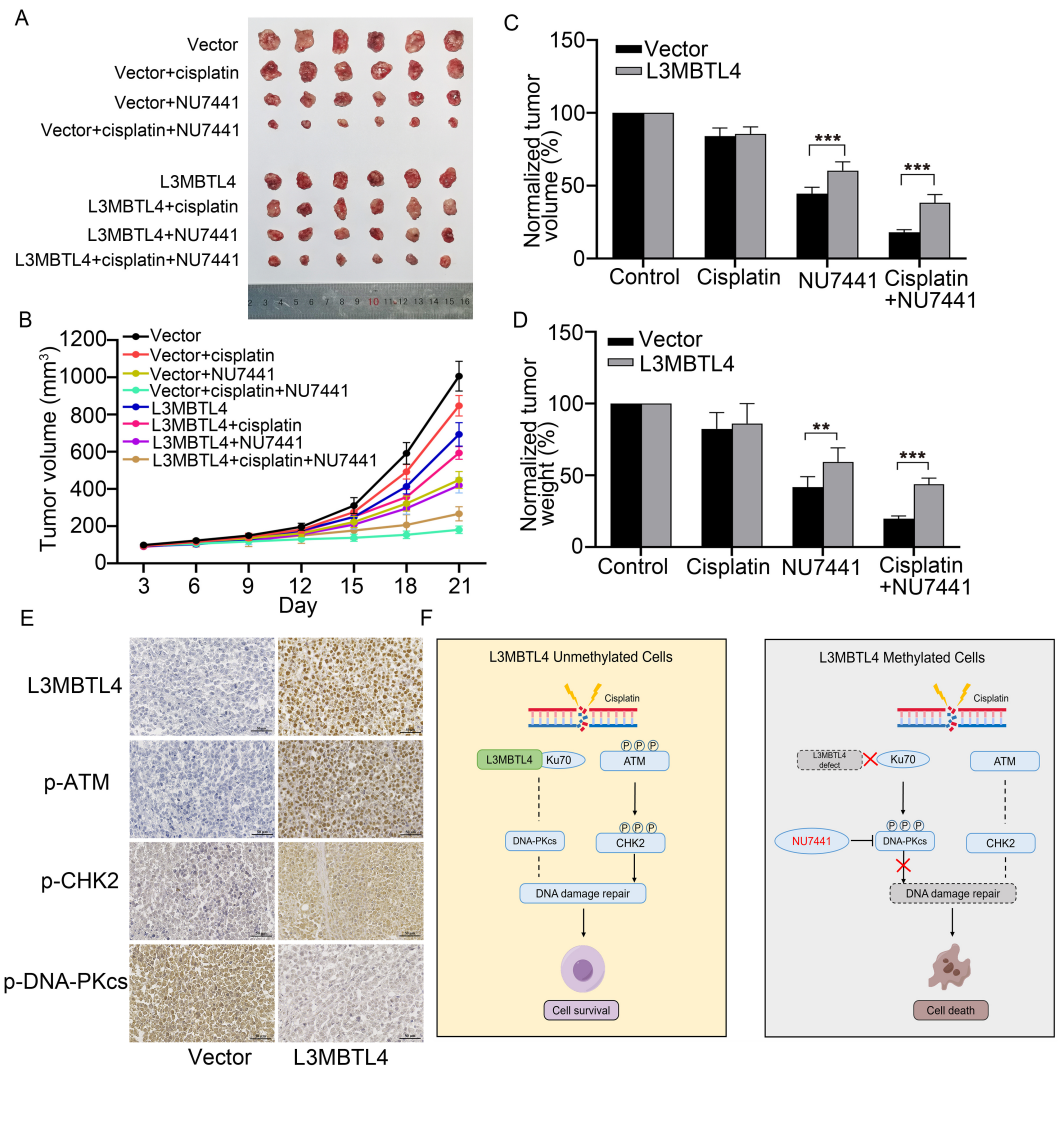
**Methylation of lethal 3 malignant brain tumor like 4 (*L3MBTL4*) sensitizes pancreatic ductal adenocarcinoma (PDAC) cell xenografts to DNA-PK inhibitor.** (**A**) Xenografts of *L3MBTL4* unexpressed and re-expressed MIA PaCa-2 cells in mice treated as indicated. (**B**) Growth curves of xenograft tumors under the treatment. (**C**) and (**D**) Normalized tumor volume and weight in *L3MBTL4* un-expressed and re-expressed MIA PaCa-2 cell xenografts. (**E**) Representative immunohistochemistry (IHC) results showing the levels of p-ATM, p-CHK2, and p-DNA-PKcs in MIA PaCa-2 cell xenografts under treatment of 2 mg/kg cisplatin. Scale bar: 50 μM. (**F**) The schematic to show epigenetic silencing *L3MBTL4* sensitizing PDAC cells to DNA-PK inhibitor. **P < 0.01, ***P < 0.001.

To further verify that *L3MBTL4* deficiency increased the sensitivity of PDAC to DNA-PK inhibitors, a MIA PaCa-2 cell xenograft mouse model was utilized. For the control group, without cisplatin or NU7441 treatment, the normalized tumor volume and weight were designated as 100%. In *L3MBTL4* silenced and re-expressed MIA PaCa-2 cell xenografts, the normalized tumor volume was 84.2 ± 5.5% vs. 85.5 ± 5.0% for cisplatin group, 44.6 ± 4.5% vs. 60.4 ± 5.9% for NU7441 group (*P* < 0.001), and 17.9 ± 1.9% vs. 38.4 ± 5.5% for combined both cisplatin and NU7441 groups (*P* < 0.001, Figure 5A–5C). The normalized tumor weights were 82.4 ± 11.4% vs. 86.2 ± 13.8% for cisplatin group, 41.7 ± 7.4% vs. 59.5 ± 9.7% for NU7441 group (*P* < 0.01), and 19.7 ± 2.0% vs. 43.9 ± 4.2% for combined both cisplatin and NU7441 groups in *L3MBTL4* silenced and re-expressed xenografts, respectively (*P* < 0.001, Figure 5D). These findings suggest that *L3MBTL4* deficiency sensitizes PDAC cells to NU7441.

To further explore the effect of *L3MBTL4* on DDR in vivo, MIA PaCa-2 cell xenograft tumors were stained by IHC. With cisplatin treatment, the levels of p-ATM and p-CHK2 were increased, while the level of p-DNA-PKcs was decreased in *L3MBTL4* re-expressed xenograft tumors, showing the promoting role in ATM/CHK2 pathway and the inhibiting role in NHEJ signaling (Figure 5E). These results suggest that epigenetic silencing of *L3MBTL4* sensitizes PDAC cells to DNA-PK inhibitors.

## Discussion

Precision medicine was mainly focused on diseases with “gain-of-function” mutations. “Loss-of-function” in cancers, caused by genetic or epigenetic abnormalities, was regarded as undruggable targets for a period of time. With the discovery of “synthetic lethality” for cancer therapy, genome wide screening for DDR gene mutations was performed with next-generation sequencing in PDAC and other cancers [41]. Unfortunately, limited “synthetic lethality” therapeutic strategies were developed based on “BRCAness” [10]. The challenge is to identify bona fide “BRCAness” tumors, and the biological consequences of each individual DDR gene mutation need to be thoroughly investigated [10, 42]. Aberrant epigenetic modifications have been recognized as the hallmarks of cancer [43]. Beyond “BRCAness”, epigenetic silencing of DDR or cell fate-determining genes may provide more opportunities for “synthetic lethality” therapeutics [37, 44–47]. In this study, *L3MBTL4* was discovered to be frequently methylated in PDAC, and methylation regulated its expression. *L3MBTL4* methylation was significantly associated with tumor size, differentiation and progression, implying that *L3MBTL4* methylation may serve as a potential diagnostic and prognostic marker. The function of *L3MBTL4* was subsequently investigated in PDAC. *L3MBTL4* suppressed cell proliferation and induced apoptosis and G1/S arrest, which implies that it plays a tumor suppressor role. The MBT domain proteins have been recognized to play crucial roles in chromatin remodeling and DDR via targeting modification of various proteins [31, 48]. Dysregulation of these proteins has been linked to cancer and other diseases [32, 33]. To better understand the mechanism of *L3MBTL4*, IP was performed. Among the proteins interacting with L3MBTL4, Ku70 was shown to have the highest score. The interaction of L3MBTL4 and Ku70 was verified by reciprocal IP and western blotting, as well as analyzed by AlphaFold3. Ku70 is a key player in NHEJ pathway, and the deficiency of DDR will increase the opportunity for gene mutations, resulting in carcinogenesis and cancer progression. On the other hand, DDR deficiency may increase the chemo-radio-sensitivity of cancer cells [49]. In addition, two of MBT domain-containing proteins (L3MBTL1 and L3MBTL2) have been reported to take part in HR [32, 33]. Therefore, we evaluated the impact of *L3MBTL4* on DDR. *L3MBTL4* was shown to reduce the sensitivity of PDAC cells to cisplatin, reflecting the potential role of *L3MBTL4* in DDR. Thereafter, the effect of *L3MBTL4* on DSB repair was tested by a comet assay, and the results revealed that *L3MBTL4* diminishes DNA DSBs in PDAC cells. Further study revealed that *L3MBTL4* activated ATM/CHK2 and inhibited NHEJ signaling pathway. Other studies have reported that ATM/CHK2 and NHEJ pathways may be balanced to be activated, supporting our findings [50–53]. To explore the potential application of *L3MBTL4* deficiency in PDAC therapy, NU7441, a DNA-PK inhibitor, was applied. The results demonstrate that silencing/deletion of *L3MBTL4* sensitizes PDAC cells to NU7441 in vitro and in vivo. *L3MBTL4* methylation may serve as a potential DNA-PK inhibitor therapeutic marker for PDAC (Figure 5F). However, there are some limitations for present model, such as potential off-target or pleiotropic effects of NU7441.

### Conclusions

In summary, *L3MBTL4* is a new component of DDR, and *L3MBTL4* methylation is a potential diagnostic and therapeutic marker of PDAC.

## Abbreviations

5-aza: 5-aza-2’-deoxycytidine
BSSQ: bisulfite sequencing
DDR: DNA damage repair
DSBs: DNA double-strand breaks
HR: homologous recombination
IC50: 50% inhibitory concentration
IHC: immunohistochemistry
IP: immunoprecipitation
IPMN: intraductal papillary mucinous neoplasm
*L3MBTL4*: lethal 3 malignant brain tumor like 4
MBT: malignant brain tumor
MCN: mucinous cystic neoplasm
MSP: methylation specific PCR
MTT: 3-(4,5-dimethylthiazol-2-yl)-2,5-diphenyltetrazolium bromide
NHEJ: non-homologous end joining
OS: overall survival
PDAC: pancreatic ductal adenocarcinoma
PI: propidium iodide

## Supplementary materials

The supplementary tables for this article are available at: https://www.explorationpub.com/uploads/Article/file/1002382_sup_1.pdf.

The supplementary figure for this article is available at: https://www.explorationpub.com/uploads/Article/file/1002382_sup_2.pdf.

The supplementary materials for this article are available at: https://www.explorationpub.com/uploads/Article/file/1002382_sup_3.xlsx.

## Declarations

### Availability of data and materials

The data in the current study are available from the corresponding author on reasonable request.

### Copyright

© The Author(s) 2026.
