# Supplementary material for: L3MBTL4 methylation is a sensitive marker of DNA-PK inhibitor in pancreatic cancer
Source: Explor Target Antitumor Ther. 2026 Jul 23;7:1002382. doi: 10.37349/etat.2026.1002382 (PMC13406882; doi:10.37349/etat.2026.1002382)
Supplement: Supplementary file 1 [file 1002382_sup_1.pdf]

Table S1. List of primer sequences.

Names	Primer sequences
L3MBTL4 RT-PCR primer	5’- GCTCCGCTGGGGATTGCTA -3’ (F)
	5’- CGTCCTGATCCAAACGCTCT -3’ (R)
GAPDH RT-PCR primer	5’-GACCACAGTCCATGCCATCAC-3’ (F)
	5’-GTCCACCACCCTGTTGCTGTA-3’ (R)
L3MBTL4 MSP primer	5’- TTTTTTAGGGAATGAATATAGAGAAGGAT -3’ (UF)
	5’- ATTCCACACCCCAAAAAACCAAATAACA -3’ (UR)
	5’- TTTTAGGGAACGAATATAGAGAAGGAC -3’ (MF)
	5’- CCACACCCCGAAAAACCGAATAACG -3’ (MR)
L3MBTL4 CDS primer	5’-TGCTCTAGAATGAAACAGCCCAACAGGAAAAGGAAGC-3’ (F)
	5’- CGCGGATCCTCATCCCCTGACTTCTTGGCCTGAG -3’ (R)
L3MBTL4 BSSQ primer	5’- TTGGGATGAAGTATGGGTGAG -3’ (F)
	5’- CATACTCCCRCCRCACCTAC -3’ (R)
L3MBTL4 siRNA	siRNA#1 5’-GCGAUGUUAAUAGCCCUUATT-3’ (F)
	siRNA#1 5’-UAAGGGCUAUUAACAUCGCTT-3’ (R)
	siRNA#2 5’-GGAGGCAACACUUCACGAUTT-3’ (F)
	siRNA#2 5’-AUCGUGAAGUGUUGCCUCCTT-3’ (R)
	siRNA#3 5’-GGGAGCAACACUGCAAGUUTT -3’ (F)
	siRNA#3 5’- AACUUGCAGUGUUGCUCCCTT-3’ (R)

MSP: methylation specific PCR; BSSQ: bisulfite sequencing; siRNA: small interfering RNA; F: forward; R: reverse.

Table S2. List of antibodies.

Name	Manufacturer	Cat No.	Antigenicity	Usage
L3MBTL4	NOVUS	NBP2-15009	Rabbit	WB 1:1000
				IP 2μg
	Proteintech	26280-1-AP	Rabbit	IHC 1:50
CyclinA2	Proteintech	18202-1-AP	Rabbit	WB 1:10000
CyclinD1	Proteintech	60186-1-Ig	Mouse	WB 1:10000
CyclinE1	Proteintech	11554-1-AP	Rabbit	WB 1:1000
CDK2	Proteintech	10122-1-AP	Rabbit	WB 1:10000
Caspase3	Proteintech	19677-1-AP	Rabbit	WB 1:500
cleaved-Caspase3	Proteintech	19677-1-AP	Rabbit	WB 1:500
Bcl-2	Proteintech	12789-1-AP	Rabbit	WB 1:2000
Bax	Proteintech	50599-2-Ig	Rabbit	WB 1:2000
ATM	ZENBIO	R23317	Rabbit	WB 1:1000
Phospho-ATM (Ser1981)	CST	58835	Rabbit	WB 1:1000
				IHC 1:400
CHK2	ZENBIO	R23920	Rabbit	WB 1:1000
Phospho-CHK2 (Thr68)	ZENBIO	340766	Rabbit	WB 1:1000
				IHC 1:400
ATR	CST	2790s	Rabbit	WB 1:1000
Phospho-ATR (Ser428)	CST	2853T	Rabbit	WB 1:1000
CHK1	ZENBIO	R380200	Rabbit	WB 1:1000
Phospho-CHK1 (Ser296)	ZENBIO	R381233	Rabbit	WB 1:1000
Ku70	Proteintech	10723-1-AP	Rabbit	WB 1:2000
				IP 2 μg
Ku80	Proteintech	16389-1-AP	Rabbit	WB 1:2000
DNA-PKcs	ZENBIO	200618-6D1	Mouse	WB 1:1000
p-DNAPKcs (Ser2056)	ZENBIO	380800	Rabbit	WB 1:1000
				IHC 1:400

γ-H2AX (Ser139)	Cell Signaling	9718	Rabbit	WB 1:1000
β-actin	Proteintech	66009-1-Ig	Mouse	WB 1:50000

IP: immunoprecipitation; WB: western blot; IHC: immunohistochemistry.
