# Supplementary material for: L3MBTL4 methylation is a sensitive marker of DNA-PK inhibitor in pancreatic cancer
Source: Explor Target Antitumor Ther. 2026 Jul 23;7:1002382. doi: 10.37349/etat.2026.1002382 (PMC13406882; doi:10.37349/etat.2026.1002382)
Supplement: Supplementary file 2 [file 1002382_sup_2.pdf]

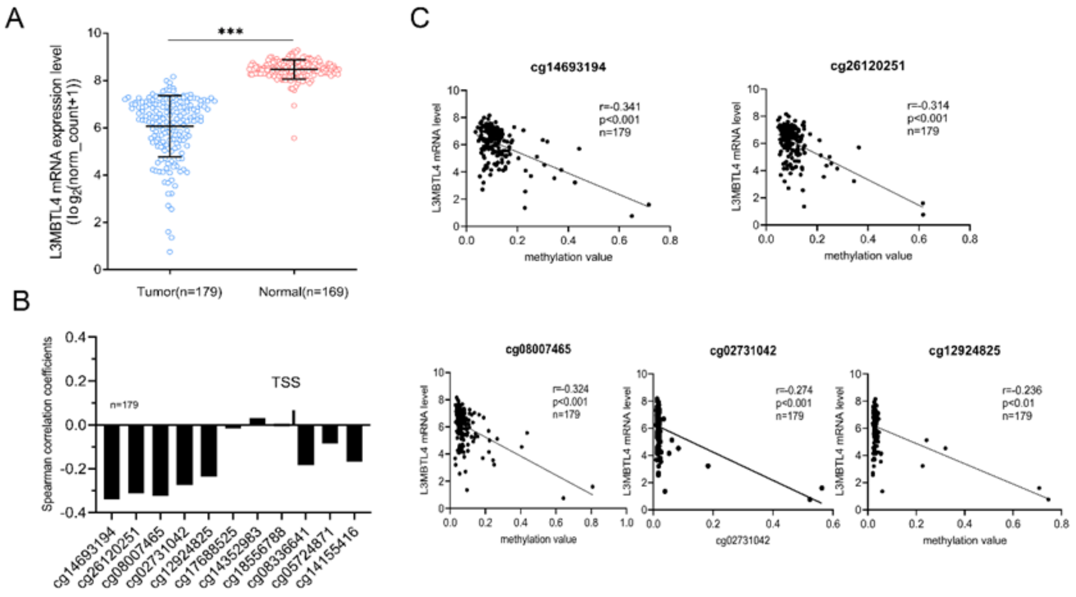

Figure S1. The expression and methylation status of L3MBTL4 in TCGA and GTEx database. (A) The expression levels of L3MBTL4 in pancreatic cancer tissue and normal tissue samples. ****P* < 0.001. (B) The coefficients of L3MBTL4 mRNA expression and methylation status of 11 CpG sites in the promoter region of L3MBTL4 by Spearman analysis. (C) Scatter plots representing a reverse association between L3MBTL4 mRNA expression and L3MBTL4 CpG sites methylation.
